# Autophagy-Related Pathways in Vesicular Unconventional Protein Secretion

**DOI:** 10.3389/fcell.2022.892450

**Published:** 2022-06-14

**Authors:** Shin Hye Noh, Ye Jin Kim, Min Goo Lee

**Affiliations:** ^1^ Severance Biomedical Science Institute, Yonsei University College of Medicine, Seoul, South Korea; ^2^ Department of Pharmacology, Brain Korea 21 Project for Medical Science, Yonsei University College of Medicine, Seoul, South Korea

**Keywords:** unconventional protein secretion (UPS), autophagy, multi-vesicular body, GRASP, golgi bypass

## Abstract

Cellular proteins directed to the plasma membrane or released into the extracellular space can undergo a number of different pathways. Whereas the molecular mechanisms that underlie conventional ER-to-Golgi trafficking are well established, those associated with the unconventional protein secretion (UPS) pathways remain largely elusive. A pathway with an emerging role in UPS is autophagy. Although originally known as a degradative process for maintaining intracellular homeostasis, recent studies suggest that autophagy has diverse biological roles besides its disposal function and that it is mechanistically involved in the UPS of various secretory cargos including both leaderless soluble and Golgi-bypassing transmembrane proteins. Here, we summarize current knowledge of the autophagy-related UPS pathways, describing and comparing diverse features in the autophagy-related UPS cargos and autophagy machineries utilized in UPS. Additionally, we also suggest potential directions that further research in this field can take.

## Introduction

Since the processes underpinning secretory protein synthesis and the intracellular trafficking pathway were initially identified over 50 years ago, it has been believed that the majority of proteins secreted into the extracellular space or to be anchored on the plasma membrane move along the classical trafficking pathway (Blobel and Sabatini, 1971; Palade, 1975; Rothman, 1994). Secretory proteins bearing a signal sequence (also known as the leader sequence) or transmembrane domain are targeted to the endoplasmic reticulum (ER) where they undergo N-glycosylation, folding, quality control, and oligomerization, followed by sorting into COPII-coated vesicles before transport to the Golgi apparatus en route to their final destinations (Ferro-Novick and Brose, 2013). In addition, it has also been noted that signal peptide-lacking proteins (leaderless proteins) and some integral membrane proteins can be secreted or reach the plasma membrane via one of several alternative routes collectively known as the unconventional protein secretion (UPS) pathway (Rabouille et al., 2012). To date, it has been revealed that a considerable number of proteins can be transported via UPS and that diverse machineries and mechanisms are involved.

**FIGURE 1:**
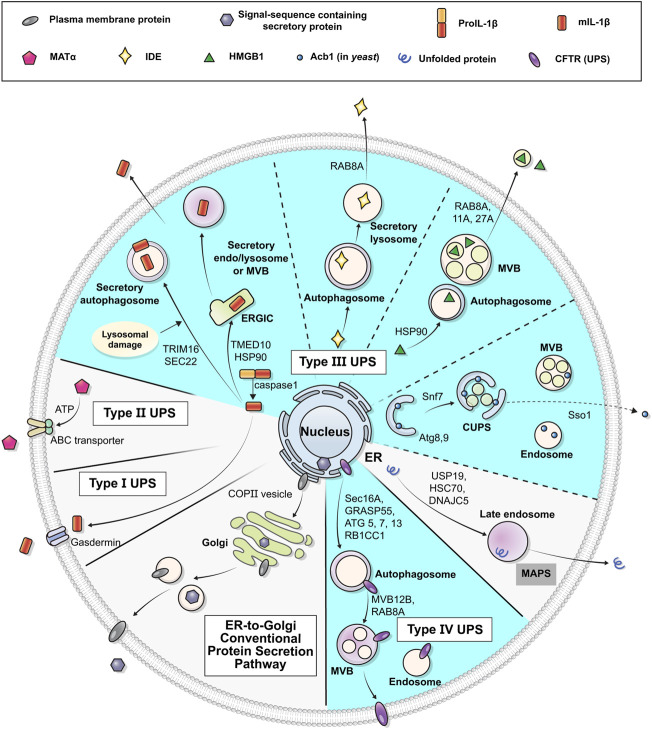
Schematic representation of unconventional protein secretion. Transmembrane proteins and ER luminal cytosolic proteins in general pass through the ER and the Golgi apparatus in their route to their final destinations (conventional secretion). Some leaderless cytosolic proteins can be secreted to extracellular space from cytosol via the membrane pore (Type I UPS), ABC-transporter (Type II UPS), or vesicles (Type III UPS). Some transmembrane proteins may alternatively be transported to the plasma membrane via an unconventional secretory pathway that bypasses the Golgi (Type IV UPS). Diverse vesicular systems, including autophagosomes, multivesicular bodies (MVBs) and CUPS, are involved in the type III and IV UPS pathways. The UPS cargos utilizing autophagy machineries are marked in cyan (see text for details).

The UPS pathways can be classified into four types according to the distinct features of the cargo proteins and mediating mechanisms involved: type I, pore-mediated translocation across the plasma membrane; type II, ABC transporter-mediated secretion; type III, membrane-bound intermediates; and type IV, Golgi bypass of transmembrane proteins (Figure 1) (Nickel and Rabouille, 2009; Rabouille et al., 2012; Rabouille, 2017). The type I, II and III UPS cargos are mostly leaderless soluble proteins, lacking a signal peptide, and are synthesized in the cytosol. On the other hand, type IV UPS cargos are originally synthesized in the ER and bypass the Golgi apparatus under certain circumstances, such as ER stress or ER-Golgi blockade, or at specific cellular localizations such as cilia and neuronal axons (Gee et al., 2018; Gonzalez et al., 2018). Whereas type I and II cargos are secreted into the extracellular space across the plasma membrane directly, type III and IV pathways utilize vesicular intermediates. These vesicular transport pathways are mediated by membrane-bound compartments, in which hydrophilic/cytosolic proteins are carried enclosed in the lumen of vesicles while integral membrane proteins are delivered via insertion into the vesicular membranes. Secretory proteins following the conventional pathway mostly use COPI, COPII, and clathrin-coated vesicles for their movement to the next stop or destination (Bard and Malhotra, 2006; Béthune and Wieland, 2018); however, the molecular nature of the vesicular machineries that mediate UPS appears to be complicated rather than composed of a single unified route. Many researchers have suggested a number of components and mechanisms that could modulate vesicular UPS. Notably, mounting evidence highlights the importance of autophagy-related proteins in the vesicular UPS.

Autophagy is an evolutionarily conserved mechanism originally defined as a degradative process which, upon starvation or cellular signal, is induced to recycle and supplement nutrients. This process involves the degradation of cytoplasmic materials (damaged organelles or dispensable components) to eliminate toxic and superfluous cytosolic substances via engulfment into the double-membranous vesicle (or so-called autophagosome), which is the most characteristic feature of this process, followed by degradation through fusion with a lysosome to ultimately maintain intracellular homeostasis (Glick et al., 2010). However, elimination of the captured material is not the only function of autophagy. Recent results show that autophagy components also play diverse roles in non-degradative processes including those occurring in the protein secretory pathways (Dupont et al., 2011; Bestebroer et al., 2013; Cadwell and Debnath, 2018; Cruz-Garcia et al., 2018; Galluzzi and Green, 2019).

The UPS process is generally activated under cellular stress conditions and is considered part of the cellular stress response (Kim et al., 2018; Park et al., 2020). Similarly, autophagy is also triggered by various cellular stress-inducing stimuli, so that the UPS and autophagy processes may share many common traits with significant overlap. There are some conceptual overlaps among UPS, secretory autophagy, and extracellular vesicle (EV) secretion. The EVs are small, membrane-bound particles secreted from cells, which can range from ∼30–1,000 nm in diameter, and the smallest type of EV is the exosome, which is ∼30–150 nm in size. While protein secretion via UPS includes the cell-surface translocation of transmembrane proteins, the secretory autophagy and exosome secretion entail the EV-mediated extracellular secretion of non-protein cargos such as RNAs and DNAs. Recent advances in our understanding of secretory autophagy and its role in EV secretion have been summarized in several reviews (Ponpuak et al., 2015; Leidal and Debnath, 2021). Here, we summarize the recent discoveries, showing where UPS and autophagy overlap and in particular focusing on the role of autophagy machineries in the vesicular type III and IV UPS pathways.

## Autophagy-Related UPS Cargos

Due to the vesicular nature of autophagy, unlike in the case of non-vesicular type I (e.g., FGF2, HIV-TAT) and type II (e.g., yeast MATα) UPS, the vesicular UPS cargos such as hydrophilic cytosolic proteins in type III UPS and transmembrane proteins in type IV UPS are subject to autophagy-related UPS cargos (Table 1).

**TABLE 1:** Autophagy-dependency of UPS cargo proteins.

	UPS type		UPS cargo	References
Non-vesicular	Type I		FGF2, HIV-TAT, IL-1β^a^, PfCDPK1, tau^a^	Chang et al. (1997); Möskes et al. (2004); Schäfer et al. (2004); Evavold et al. (2018); Merezhko et al. (2018)
	Type II		MATα, HASPB	McGrath and Varshavsky, (1989); Denny et al. (2000)
Vesicular	Type III	Autophagy- related	AcbA, Acb1, IL-1β^a^, ferritin, IL18, HMGB1, MMP2, TGM2, α-synuclein^b^, IDE, FABP4^b^, α-crystallin-B/HSPB5, TGFβ1, PARK7/DJ-1, annexin-A2, tau^a^, SOD1	Kinseth et al. (2007); Duran et al. (2010); Manjithaya et al. (2010); Dupont et al. (2011); Poehler et al. (2014); Son et al. (2016); Chen et al. (2017); Kimura et al. (2017); Nüchel et al. (2018); Urano et al. (2018); Josephrajan et al. (2019); Kang et al. (2019); D'Agostino et al. (2019); Nüchel et al. (2021)
		Not-defined	tau^a^, LIF, FAM3C, DKK3, AIM2, ARMS2, BAG3	
	Type IV	Autophagy- related	CFTR, Mpl	George et al. (1999); Baldwin and Ostergaard, (2002); Hasdemir et al. (2005); Penuela et al. (2007); Schotman et al. (2008); Merregaert et al. (2010); Hoffmeister et al. (2011); Gee et al. (2011); Tian et al. (2014); Cleyrat et al. (2014); Jung et al. (2016); Gee et al. (2018)
		Not-defined	αPS1 integrin in *drosophila*, polycystin2, M2 mutant of smoothened, Peripherin/rds,CD45, Connexin26 and 30, Kv4, pannexin1 and 3, phospholipid scramblase1, pendrin	

a Following diverse routes.

b Existing contrary results.

### Cytokines

It has been shown that several cytokines that do not bear the leader sequence are secreted in an autophagy-dependent manner. For example, interleukin-1 (IL-1) family members are secreted without entering the ER-Golgi conventional pathway after processed from their precursors upon inflammasome activation (Dupont et al., 2011). Secretion of IL-1β is enhanced upon stimulation of autophagy by the starvation and nigericin co-treatment, which depends on ATG5, GRASP55, and Rab8a (Dupont et al., 2011). High mobility group box 1 (HMGB1) and IL-18 also are released in a similar manner. HMGB1 is a nuclear protein whose secretion is mediated by inflammasome in response to various stimuli. Secretion of HMGB1 is inhibited by an early autophagy inhibitor, wortmannin or 3-methyladenine (3-MA), and reduced in ATG5-deficient cells (Kim et al., 2021; Wang et al., 2021).

Moreover, signal peptide-bearing proteins can be released via UPS under certain conditions. For example, transforming growth factor beta 1 (TGFβ1), a multifunctional cytokine possessing a signal peptide and playing a role in apoptosis, differentiation and development, was proposed to be secreted via different pathways independent of the conventional route while also involving the autophagic components (Nüchel et al., 2018). In addition, it has been indicated in a proteome analysis that approximately 40–50% of mTORC1- and GRASP55-dependent secretome and surfactome proteins harbor a signal peptide, implying that many signal peptide-bearing cargos can use UPS pathways for their delivery to the surface under certain cellular conditions (Nüchel et al., 2021). Further investigation is required to confirm whether all of these are exclusively UPS cargos, since it is possible that some mTORC1- and GRASP55-dependent secretory proteins may also undergo conventional pathways.

### Neurodegenerative Disease-Associated Proteins

A number of neurodegenerative disease-associated proteins can be delivered to the plasma membrane through autophagy-mediated UPS pathways (Ejlerskov et al., 2013; Nilsson et al., 2013). α-Synuclein, particularly in its aggregated forms, has been implicated in the pathogenesis of Parkinson’s disease (PD) and other neurological disorders (Polymeropoulos et al., 1997; Kahle, 2008). The extracellular secretion of α-synuclein may result in a cell-to-cell transmission of protein aggregates, which occurs in many neurodegenerative disorders (Lee et al., 2005; Lee et al., 2010). Several studies have indicated that α-synuclein undergoes an autophagy-related UPS pathway (Ejlerskov et al., 2013; Cleyrat et al., 2014; Poehler et al., 2014; Yang et al., 2017). However, the role of autophagy in α-synuclein secretion has also been challenged by other researchers whose studies suggest that autophagy inhibition could promote α-synuclein secretion (Lee et al., 2013; Lee and Lee, 2016), and thus further investigation is required for clarification of specifically which autophagy components mediate α-synuclein secretion and how they do so. Accumulation of misfolded aggregated tau proteins in the nervous system is a hallmark of tauopathies such as Alzheimer’s disease (AD). The tau secretion through the UPS pathway involves both non-vesicular and vesicular mechanisms (Brunello et al., 2020); and several recent studies suggest that autophagy machinery participates in the vesicular route-mediated tau secretion (Mohamed et al., 2014; Kang et al., 2019; Chen et al., 2020).

It is noteworthy that proteins which inhibit the aggregation of misfolded proteins, by promoting degradation or assisting the proper folding of aggregation-prone proteins, can also be secreted via an autophagy-associated UPS route. PARK7/DJ-1 (Parkinsonism associated deglycase) is a PD- and cancer-associated protein devoid of the leader sequence, functioning as a redox-sensitive chaperone and protein deglycase that inhibits the aggregation of α-synuclein and protects neurons against oxidative stress and cell death. Autophagy-related proteins ATG5, ATG9, and ATG16L1 were shown to be required for PARK7 secretion (Urano et al., 2018). Insulin-degrading enzyme (IDE) is a ubiquitously expressed protease with the ability to degrade pathological extracellular substrates such as an amyloid-beta peptide (Aβ), whose accumulation is associated with AD. IDE secretion has been reported to be unaffected by brefeldin A (BFA), an inhibitor of the conventional secretion pathway (Zhao et al., 2009), suggesting relevance to an unconventional secretion pathway; and other results, showing that a deficiency of ATG7 in mice decreases IDE activity, provide further validation of the role of IDE as a cargo of the autophagy-based UPS pathway (Son et al., 2016). α-Crystallin B (or HspB5) is a small heat-shock chaperone preventing aggregation and unfolding of target proteins under conditions of cellular stress and requires the autophagy-related unconventional pathway (D'Agostino et al., 2019). Mutant huntingtin (mHTT), causing Huntington’s disease, has also been suggested as a cargo for autophagy-dependent UPS (Ahat et al., 2021). Mutant HTT secretion is enhanced under cellular stress conditions which involve autophagy activations including ATG7. Lastly, the mutant form of superoxide dismutase 1 (SOD1), linked to amyotrophic lateral sclerosis (ALS), has been shown to be secreted via a nutrient starvation-induced UPS pathway in yeast and HeLa cells (Cruz-Garcia et al., 2017).

### Lipid Binding Proteins

Several lipid-binding proteins are shown to be secreted via an autophagy-associated UPS pathway. Annexin A2 (ANXA2), a phospholipid binding protein, was observed in autophagosomes and multivesicular bodies (MVBs), followed by exosomal release, but not in ATG5 knockdown cells (Chen et al., 2017). Fatty acid binding protein 4 (FABP4) is a signal sequence-lacking protein and mainly expressed in adipocytes to regulate the transport of lipids. FABP4 has been proposed to be a UPS cargo, but was initially described as an autophagy-independent cargo because FABP4 secretion was not affected in ATG5 deficient cells (Villeneuve et al., 2018). However, a later study showed that early autophagic genes such as Ulk1/2, Fip200 or Beclin-1 were required for FABP4 secretion even though knockdown of ATG5 enhanced the FABP4 release (Josephrajan et al., 2019).

### Proteins in Yeast

Autophagy has been extensively investigated in yeast (Takeshige et al., 1992; Ohsumi, 2014), with early reports attempting to delineate the autophagy-related UPS cargos in yeast (Duran et al., 2010; Manjithaya et al., 2010). Secretion of Acb1 in yeast is induced by rapamycin, an autophagy activator, and is mediated by core autophagy machinery proteins which are necessary for autophagosome formation. Malhotra et al. identified a membrane structure in yeast, known as the compartment for unconventional protein secretion (CUPS), as being the source of organelles or trafficking intermediates for autophagy-based unconventional secretion of Acb1 (Bruns et al., 2011). It has been suggested that the CUPS is built during starvation and formed by COPI-independent extraction of membranes from the early Golgi cisternae, requiring PI4P for its biogenesis and PI3P for stability (Cruz-Garcia et al., 2014).

### Transmembrane Proteins

Another important class of autophagy-related UPS is the type IV UPS whose substrates are transmembrane proteins. Despite harboring a signal peptide, a number of transmembrane proteins (e.g., CFTR, Mpl, Pendrin, αPS1 integrin) have been found to bypass the Golgi when reaching the plasma membrane under specific conditions (Schotman et al., 2008; Hoffmeister et al., 2011; Tian et al., 2014; Jung et al., 2016; Gee et al., 2018). Among these, two cargos have hitherto been proven to be associated with autophagy: cystic fibrosis transmembrane regulator (CFTR) and myeloproliferative leukemia protein (Mpl) (Table 1).

*N*-glycosylated membrane proteins acquire their complex glycosylation pattern while they travel through the Golgi (Reily et al., 2019). Golgi-mediated complex-glycosylation is thus considered to be a feature of proteins undergoing the conventional pathway, whereas proteins bypassing the Golgi demonstrate only an immature form of ER core-glycosylation. Therefore, the cell-surface expression of ER-core glycosylated proteins can be a sign of Golgi-bypass for those transmembrane proteins for which Golgi-mediated glycosylation patterns are defined, including CFTR, pendrin, and Mpl. CFTR is an anion channel transporting Cl^−^ and HCO_3_^−^ across the apical membrane of epithelial cells and its mutations cause cystic fibrosis (CF) and chronic pancreatitis (Lee et al., 2012; Kim et al., 2020). The most common CFTR mutation among CF patients is the deletion of phenylalanine at the amino acid position 508 (∆F508-CFTR), resulting in folding defects followed by ER-associated degradation (ERAD). Therefore, the folding defective ∆F508-CFTR neither exits the ER nor reaches the plasma membrane via the conventional secretory pathway. Gee et al. (2011) have shown that the ER core-glycosylated ∆F508-CFTR can be rescued to the cell surface via a UPS pathway bypassing the Golgi, this process being induced by ER stress or GRASP55 overexpression in both mammalian cells and mouse models. Later studies have indicated that the early and core machineries of autophagy are required for the transport of ∆F508-CFTR to the plasma membrane via the UPS route (Noh et al., 2018). The core-glycosylated ER form of Mpl (a.k.a. the thrombopoietin receptor) has similarly been shown to reach the cell surface via a non-canonical pathway, involving the mechanism taken up into LC3-positive autophagic structures (Cleyrat et al., 2014). The UPS both of CFTR and Mpl utilizes GRASP55 for translocation of the proteins to the plasma membrane in addition to the autophagy machineries (Cleyrat et al., 2014; Noh et al., 2018).

## Autophagy Machineries Utilized in UPS

### Autophagy-Related Genes and Proteins

Autophagy can be subclassified into three types, depending on the manner of cargo delivery to the lysosome: macroautophagy, microautophagy, and chaperone-mediated autophagy (CMA) (Mizushima et al., 2011). Among them, macroautophagy is the major type of autophagy and the most extensively researched. Macroautophagy is defined as a mechanism reliant on the ordered processing of ATG proteins congregated at the phagophore assembly site (PAS) to construct a double membranous organelle autophagosome, which will fuse with a lysosome. The overall macroautophagy (hereafter referred to as autophagy) process is composed of multiple steps: 1) initiation by stimuli inducing autophagy; 2) nucleation of the membrane, formed from diverse membrane sources, at the PAS; 3) expansion and formation of the double membrane structure with engulfment substrates; 4) fusion with lysosomal compartments; and 5) degradation of the captured materials.

The Autophagy-related genes (ATGs), evolutionarily conserved in mammals (Mizushima, 2018), were initially discovered in yeast and are known to be required for nucleation and elongation during the biogenesis of autophagosome (Klionsky, 2007). Recent evidence indicates that many ATG proteins perform a role not only in classical degradative autophagy but also in membrane trafficking, exosome secretion, and UPS (Galluzzi and Green, 2019). ATG5 is a representative protein participating in the early stages of the autophagy process. A knockdown or knockout ATG5 model has been utilized in most articles suggesting the association of UPS with autophagy (Dupont et al., 2011; Gee et al., 2011; Cleyrat et al., 2014; Urano et al., 2018; Kim et al., 2021). However, ATG5 does not seem to be involved in the UPS processes of some cargo proteins such as ANXA2 and FABP4. For example, as aforementioned, secretion of FABP4 from white adipose tissue upon lipolytic stimulation requires components of the ULK complex and PI3K class III that are necessary for autophagy initiation, while ATG5 inhibition enhances the FABP4 release (Josephrajan et al., 2019).

LC3B, an ATG8 family member, is most widely used as an autophagy marker. The conversion of LC3B-I into LC3B-II represents autophagosome maturation, since ATG8 proteins should become lipidated to be anchored in the autophagosome membranes and lipidated ATG8 proteins can recruit LIR-containing substrates (Klionsky et al., 2021). Deletion or knockdown of ATG8 genes have been shown to result in a reduced secretion of Acb1 and CFTR (Manjithaya et al., 2010; Gee et al., 2011). Quantitative measurements of lipidated LC3B-II relative to LC3B-I have been examined in several studies to show the relevance of autophagy in the UPS of diverse cargo proteins (Son et al., 2016; Noh et al., 2018; Urano et al., 2018; Josephrajan et al., 2019; Wang et al., 2021).

Not only ATG5 and LC3, but also multiple ATG proteins are involved in UPS. In yeast, Atg1, Atg6, Atg8, Atg9, Atg11, and Atg17 are necessary for Acb1 secretion in P. *pastoris* (Manjithaya et al., 2010). Additionally, Atg5, Atg7, Atg8 and Atg12 have been shown to be required for Acb1 secretion in S. *cerevisiae* (Duran et al., 2010). ATG16L1, interacting with the ATG12-ATG5 conjugate, also participates in the UPS of IL-1β, PARK7/DJ-1, and α-synuclein in mammalian cells (Kimura et al., 2017; Urano et al., 2018; Burbidge et al., 2021). The respective secretions of chemokine C-X-C motif ligand 8 (CXCL8), leukemia inhibitory factor (LIF), and family with sequence similarity 3 member C (FAM3C) were each enhanced when low-autophagy melanoma cells were treated with the autophagy-inducing tat-BECN1 peptide and reduced when ATG7 was silenced in high-autophagy cells (Kraya et al., 2015). The only autophagy related integral membrane protein, ATG9 (yeast Atg9), is proposed to be a supplier of autophagic membrane sources for the UPS of Acb1 and PARK7/DJ-1 (Manjithaya et al., 2010; Bruns et al., 2011).

Since phosphoinositide 3-kinase class 3 (PI3KC3) plays a key role in regulating autophagosome formation, the decrease in cargo secretion resulting from inhibition of PI3KC3 is also indicative of the relationship between autophagy and UPS. Not only genetic silencing of PI3KC3 itself, but also pharmacological inhibitory reagents (e.g., 3-MA, wortmannin, and Vps34-IN136) were utilized in an effort to establish the autophagic dependency of various UPS processes (Son et al., 2016; Noh et al., 2018; Nüchel et al., 2018; D'Agostino et al., 2019; Josephrajan et al., 2019; Kim et al., 2021).

### Autophagy Receptors and Adaptors

Although classical autophagy degrades cytoplasmic materials non-selectively, each UPS cargo seems to use different autophagy machineries. An explanation of precisely how cargo selection occurs, mechanistically, could perhaps be found in the cargo receptors involved in selective autophagy (Kirkin and Rogov, 2019). During selective autophagy, the cargo proteins, subsequent to being labeled with ubiquitin or galectins, are recognized by autophagic receptors such as SQSTM1, NBR1 and CALCOCO2 and in turn link to autophagosomal membranes (Johansen and Lamark, 2020). It has been shown that the secretory autophagy cargo IL-1β is recognized by the autophagy cargo receptor TRIM16 before interacting with the Sec22b to facilitate the delivery of IL-1β to the LC3B-II positive membrane (Kimura et al., 2017). TRIM16 is known to interact with GABARAP yet not with sequestosome-1/p62 (Mandell et al., 2014), a classical degradative autophagy receptor (Bjørkøy et al., 2005), which suggests that certain receptor/adaptor proteins potentially guide cargos to the secretory route avoiding disposal mechanism. A recent study additionally shows that the galectin3-mediated UPS of α-synuclein is dependent on TRIM16 (Burbidge et al., 2021).

Because the ER membrane is regarded as the prime source of autophagy-associated membrane structures, adaptor proteins in the ER-phagy (e.g., FAM134B, TRN3L, and ATL3) (Chino and Mizushima, 2020) may play a major role in the membrane vesicle formation and cargo selection in autophagy-associated UPS. Further investigation into precisely which autophagy receptors and adaptors facilitate the secretion for each cargo will shed more light on the precise mechanisms underpinning the UPS.

### SNAREs and Rab Proteins

The primary role of SNARE [soluble N-ethylmaleimide-sensitive fusion (NSF) attachment protein receptor] proteins is to mediate the fusion of vesicles with the target membrane and other membrane-bound compartments. A number of reports have indicated that SNAREs also mediate membrane fusion in autophagy-related vesicular trafficking (Moreau et al., 2013). In yeast, the plasma membrane target SNARE (t-SNARE) Sso1 and a phospholipase D (Spo14) have been shown to be required for translocation of Acb1 onto the plasma membrane via UPS (Duran et al., 2010; Manjithaya et al., 2010). The vesicular R-SNARE Sec22b and the plasma membrane Q-SNAREs syntaxin 3 and syntaxin 4 cooperate in IL-1β secretion (Kimura et al., 2017). On the other hand, VAMP7, an R-SNARE required for heterotypic fusion of autophagosomes with lysosomes, shows pleiotropic aspects in autophagy-related UPS (Gee et al., 2011; Villeneuve et al., 2018) and have a relevance linked to that of lysosome-dependency (see below).

The Rab family of proteins, moreover, which are part of the Ras superfamily of small G proteins regulating intracellular vesicular transport, are involved in autophagy-associated UPS. Examples of this are that secretory FABP4 has been localized to structures that are positive for Rab7 (Josephrajan et al., 2019) and that the UPS of TGFβ1, ANXA2 and CFTR has been shown to require RAB8A yet not RAB8B, the latter being involved in the maturation of degradative autophagosomes (Noh et al., 2018; Nüchel et al., 2018). RAB27A is a necessary requirement for the autophagy-related secretion of ANXA2, while RAB27B is not (Chen et al., 2017). Further investigations into SNAREs and Rabs may contribute to our understanding of the detailed mechanisms involved in autophagy-related UPS.

## Structural and Functional Associations Between Autophagy and UPS

### Autophagosome

Autophagosomes, which are double-membraned sequestering vesicles formed by distinct molecular components, constitute a representative morphological hallmark of macroautophagy. Therefore, the presence of UPS cargos in autophagosome-like structures has frequently been highlighted with the aim of demonstrating autophagy dependency in the UPS. Since an autophagosome can be recognized by its morphological features, an electron microscopic examination is one of the best approaches to analyze whether the UPS cargo is intermediated via autophagosomes. In practice, however, this can prove rather difficult because the autophagosome is a transient organelle in the autophagic process (Klionsky et al., 2021). The Mpl protein has been shown to be located in autophagosome-like structures during its UPS (Cleyrat et al., 2014). It has additionally been proposed that there is an association between autophagosomes and UPS-mediated CFTR trafficking to the plasma membrane via the Golgi-bypassing pathway by showing that GRASP55, a binding partner of CFTR upon UPS, has been captured in autophagosomes under UPS-evoking conditions (Noh et al., 2018).

LC3, a mammalian homolog of yeast Atg8, is abundant at both the inner and outer membranes of autophagosomes and is regarded as a reliable indicator of autophagosome presence (Tanida et al., 2008). Images showing colocalizations of LC3 and UPS cargo, via immunocytochemistry or co-fractionation on the same membrane compartments, have been described as evidence for an autophagosome-mediated process in UPS (Duran et al., 2010; Dupont et al., 2011; Zhang et al., 2015; Son et al., 2016; Chen et al., 2017; Nüchel et al., 2018; Urano et al., 2018; D'Agostino et al., 2019; Wang et al., 2021; Kim et al., 2021). However, a degree of caution is needed when employing LC3 as an indicator of autophagosomes since it can also be a component of phagophore structures other than autophagosomes (Runwal et al., 2019).

### MVB and Endosome

There is abundant evidence that autophagosomes fuse with endosomes, after the formation of MVBs and further amphisomes in mammalian cells (Liou et al., 1997; Jäger et al., 2004; Morvan et al., 2009; Razi et al., 2009). The biogenesis of MVBs via invagination of the endosomal membrane is catalyzed by the endosomal sorting complexes required for transport (ESCRT) proteins (Hurley, 2008). The MVBs and amphisomes are known to be directed to the disposal pathway by fusion with lysosomes; but recent discoveries highlight that they also play a role in the delivery of vesicular cargos to the plasma membrane, as in the case of exosome secretion (Hessvik and Llorente, 2018; Ganesan and Cai, 2021). Some UPS cargos such as IL-1β (Zhang et al., 2015) and CFTR (Noh et al., 2018) have likewise been shown to be present in MVBs interlinked with autophagy, which are routed to the extracellular space or the plasma membrane. MVB12B, a component of the ESCRT-I complex, participates in the MVB-associated Type IV UPS of CFTR (Noh et al., 2018). In yeast, Acb1 secretion also requires Vps23 and several ESCRT-I, -II, and –III, but not Vps4 for the biogenesis of CUPS (Curwin et al., 2016).

Endosomes have also been shown to be involved in the vesicular UPS. For example, the endosome-specific t-SNARE Tlg2 is required for Acb1 secretion (Duran et al., 2010), suggesting that a certain endosomal compartment serves as an intermediate structure in the UPS of Acb1. Therefore, it is plausible that vesicular structures originating from autophagosomes and MVBs may fuse with the recycling endosomes at a later stage of the UPS for the final delivery of cargo proteins to the plasma membrane. Interestingly, endosomes can also directly mediate UPS unrelated to autophagy. In a study suggesting that FABP4 secretion is independent of autophagy and MVB, endosomal compartments are required for the UPS of FABP4 (Villeneuve et al., 2018).

Interestingly, certain cytosolic misfolded proteins have been shown to be secreted via late endosomes in a process termed MAPS (misfolding-associated protein secretion), in which the USP19-DNAJC5 chaperone cascade appears to play an important role (Lee et al., 2016). In addition, it has been reported that secretion of some neurodegenerative disease-associated proteins such as Tau, SOD1, spinocerebellar ataxia 3 (SCA3), TDP43 and α-synuclein are also engaged in USP19-dependent MAPS (Xu et al., 2018). Despite MAPS and CMA being similar in that both involve HSC70 and endo-lysosomes, the latter process is enhanced upon serum starvation while the former is inhibited under the same serum deprived condition (Lee and Ye, 2018).

### CUPS

Another interesting structural compartment related to uncanonical secretion is CUPS (Bruns et al., 2011). Unconventional secretion of Acb1 in yeast is mediated by CUPS requiring Atg and MVB components, such as Sso1, Grh1, Vps23, Atg8, and Atg9. Interestingly, the CUPS formation is induced by starvation but not by rapamycin, a classical autophagy inducer. The formation of the CUPS structure requires ESCRT-I, -II and -III components, but not those of Vps4. An ESCRT-III component, Snf7, localizes to CUPS containing Grh1 (yeast GRASP) and plays a key role instead of Vps4 in the formation and stabilization of functional CUPS (Curwin et al., 2016). CUPS has hitherto only been demonstrated in yeast, and not in mammalian cells.

### Lysosome

Canonical autophagy drives autophagosome–lysosome fusion for substrate clearance. However, proteins to be secreted to the exterior of the cell via UPS need to maintain an intact form and avoid degradative processes. In order to do so, in their route to the plasma membrane, the UPS cargos are required to circumvent lysosomes, or to escape the lysosome-mediated degradation. It has been demonstrated that Acb1 in yeast is sorted for packing into autophagosomes, the outcome being extracellular release rather than degradation in lysosomes/vacuoles (Duran et al., 2010; Manjithaya et al., 2010). The plasma membrane rescue of CFTR-∆F508 via UPS is evidently unaffected by treatment of the lysosomal vacuolar H^+^ ATPase (V-ATPase) inhibitor bafilomycin A1 (Noh et al., 2018), or depletion of the vesicular SNARE for lysosomal membrane fusion VAMP7 (Gee et al., 2011). It has been shown that IL-1β secretion is not diminished by knockdown of syntaxin 17, a SNARE for autophagosome membrane fusion with the lysosome membrane (Kimura et al., 2017). In studies on α-Crystallin B UPS, the cargo-containing LC3-positive compartments were not colocalized with LAMP1, an endolysosomal marker, suggesting that they bypass lysosomes (D'Agostino et al., 2019). The bafilomycin A1 and chloroquine treatments to inhibit autolysosome formation have been shown to promote HMGB1 secretion (Kim et al., 2021). Considered collectively, these results indicate that the abovementioned cargos undergo UPS independent of lysosomes.

Conversely, the UPS of certain cargos requires structural or functional lysosome integrity. Studies have shown that when lysosomes are disrupted by bafilomycin A1, IDE (Son et al., 2016), TGFβ1 (Nüchel et al., 2018) and mHTT (Ahat et al., 2021) secretions are blocked or reduced. Interestingly, bafilomycin A1 diminished IL-1β secretion in cells stimulated for autophagy via starvation, whereas no change was observed in cells undergoing basal autophagy (Dupont et al., 2011). Mpl was shown to be colocalized with the lysosomal marker LAMP1 as well as the autophagy marker LC3, and bafilomycin A1 led to a decreased cell-surface level of immature core-glycosylated Mpl (Cleyrat et al., 2014). The knockout of VAMP7 and syntaxin 7, SNAREs involved in lysosomal membrane fusion, has been shown to reduce FABP4 secretion, suggesting the involvement of lysosomal exocytosis in the FABP4 release to the extracellular space (Villeneuve et al., 2018). These cargos may have specific mechanisms to avoid degradation during their stay at the lysosome or lysosome-like structures. For example, it has been suggested that the SlyX domain (EKPPHY) of IDE contributes to the Aβ-induced IDE secretion by preventing lysosomal degradation (Son et al., 2016).

### ERGIC and TMEDs

In addition to several cellular organelles that have been implicated as the membrane sources for autophagy (Nakatogawa, 2020), such as the ER, mitochondria, plasma membrane and Golgi, the ER-Golgi intermediate compartment (ERGIC) has also been suggested as a membrane source for autophagosome biogenesis (Ge et al., 2013) with articles suggesting associations between ERGIC and secretory pathways including autophagy-derived UPS (Bernard and Klionsky, 2014; Ge et al., 2015; Zhang et al., 2020).

The transmembrane emp24 domain-containing proteins (TMEDs, also known as p24 proteins) are a family of type I membrane proteins distributed in the membranes of the early secretory pathway. Although TMED proteins have been suggested to function as cargo receptors for the anterograde transport of certain secretory cargos (Schimmoller et al., 1995) or as primary receptors for the small GTPase of COPI-vesicle formation (Contreras et al., 2004), most of their functions remain elusive. Recently, TMED10 was identified as a translocator for IL-1β into the ERGIC, potentially via the formation of a homomultimeric channel during the UPS process (Zhang et al., 2020). However, this result is somewhat in conflict with the claim in a previous report that IL-1β is captured by the secretory autophagy receptor TRIM16 then translocated directly to the autophagosomal vesicles (Kimura et al., 2017). In addition, it has been suggested that TMED proteins in general function as stable heteromeric complexes rather than possessing a homomultimeric form (Pastor-Cantizano et al., 2016). In fact, we recently found that the heteromultimeric TMED complex cargo recruitment in the ER stress-associated type IV UPS of CFTR, pendrin, and SARS-CoV-2 Spike. More specifically, TMED3 initially recognizes the ER core-glycosylated transmembrane protein cargos; and the TMED2/3/9/10 heteromultimeric complex facilitates the UPS of these membrane cargos (Park et al., (2022)). However, the study of Zhang et al., (2020) examined neither the relationship between TMED10 and other TMEDs nor the role of other TMED proteins in IL-1β UPS. Further investigations are required to identify the mechanistic details underlying TMED10-mediated IL-1β UPS as well as the role of ERGIC as a membrane source in autophagy-associated UPS.

### mTOR and Rapamycin

The mammalian target of rapamycin (mTOR), a serine/threonine kinase, regulates autophagy by sensing cellular stresses and growth factor signals. The inhibition of the mTOR signaling pathway in general induces autophagy processes (Kim and Guan, 2015). The mTOR inhibitor rapamycin is a well-known classical autophagy inducer and is widely used to examine the autophagic dependency of cellular events. Interestingly, treatments with rapamycin have evoked variable results in the autophagy-associated UPS of different cargo proteins. The rapamycin application to activate autophagy has resulted in an increased IDE secretion from astrocytes (Son et al., 2016) and α-Crystallin B secretion from the COS-7 monkey kidney fibroblast cells (D'Agostino et al., 2019), indicating that the UPS of these cargos is dependent on the mTOR pathway. It has been demonstrated that the inactivation of the mTOR complex C1 (mTORC1) by cellular stress also affects the composition of extracellular secretome mediated via a GRASP55-dependent UPS (Nüchel et al., 2021).

In contrast, rapamycin treatments neither stimulated PARK7 release (Urano et al., 2018) nor affected CFTR UPS (Noh et al., 2018). Since the mTOR senses cellular nutritional status and is primarily involved in the induction of degradative autophagy, it is plausible that the mTOR-independent signaling pathway is more responsible for secretory autophagy. Interestingly, while rapamycin triggered Acb1 secretion in yeast (Manjithaya et al., 2010), rapamycin alone was not sufficient to induce the biogenesis of CUPS believed to play a critical role in Acb1 secretion (Bruns et al., 2011). Further investigation is needed to dissect the role of mTOR pathways in autophagy-related UPS.

### GRASP

Despite the Golgi reassembly stacking proteins (GRASPs) 55 and 65 initially being identified as components of the Golgi-stacking machinery binding to the vesicle docking protein receptor GM130 (Barr et al., 1998; Shorter et al., 1999), later research has revealed that GRASPs (Grh1 in yeast, dGRASP in *Drosophila*) are required for Golgi-independent UPS. This can be seen, for example, in the cases of the starvation-induced secretion of Acb1 (or AcbA) in yeast (Kinseth et al., 2007; Duran et al., 2010; Manjithaya et al., 2010) and the mechanical stress-induced secretion of *Drosophila* integrins (Schotman et al., 2008). Thereafter, in mammalian cells, diverse proteins following UPS, including transmembrane proteins as well as leaderless proteins, were indicated to be dependent on GRASPs for secretion (Gee et al., 2011; Son et al., 2016; Kim et al., 2020; Kim et al., 2021; Nüchel et al., 2021). In these results, GRASP55/GORASP2 was commonly identified in CUPS and autophagosome-like structures; and this observation is corroborated by recent studies determining that GRASP55 can interact with LC3 via a LIR motif in the PDZ2 domain of GRASP55 (Nüchel et al., 2018; Zhang et al., 2018).

**FIGURE 2:**
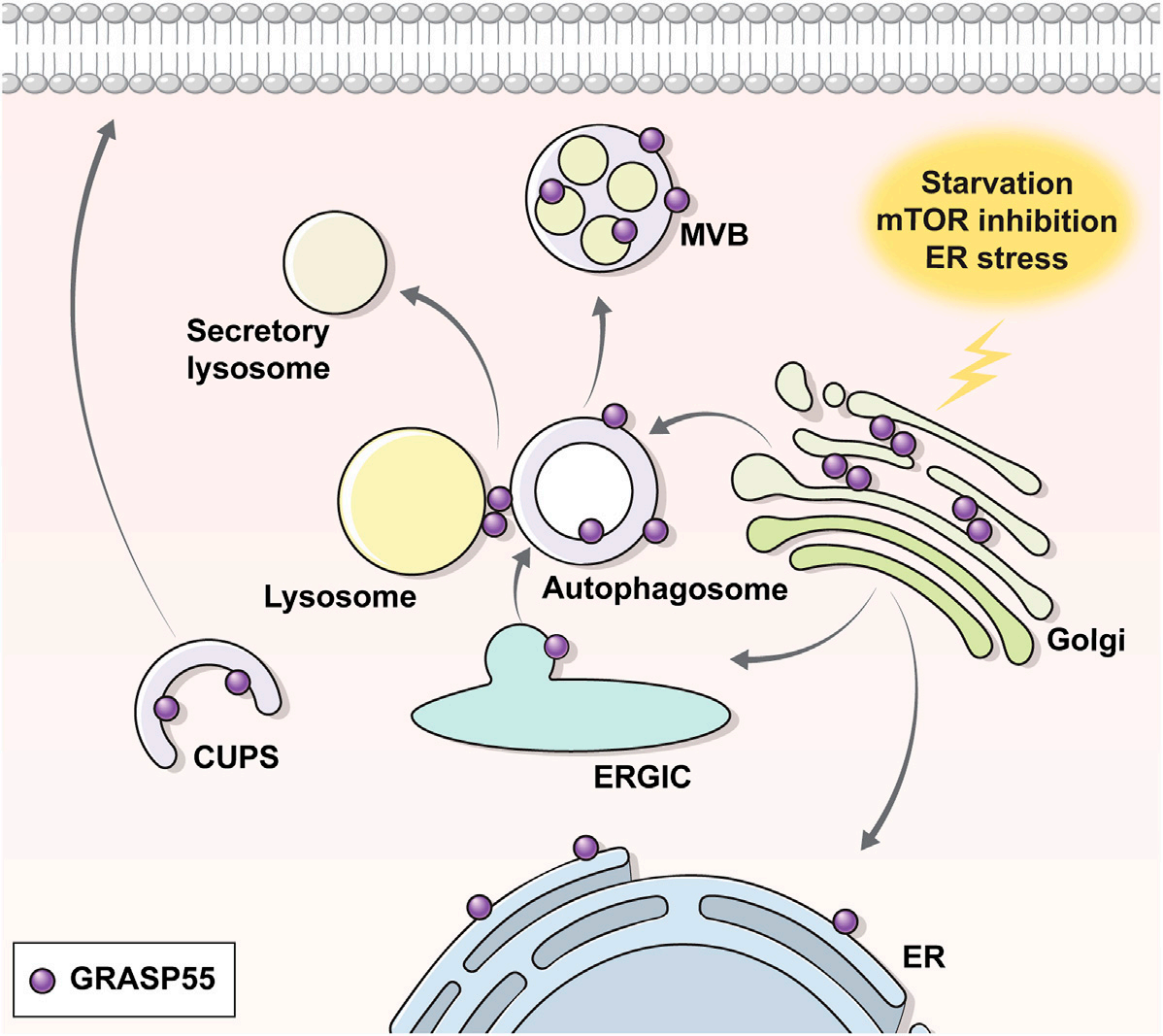
Translocation of GRASP55 upon UPS. Under normal conditions, the Golgi peripheral protein GRASP55 resides at the Golgi. Under UPS-inducing conditions (e.g., starvation, ER stress, ER-to-Golgi block etc.), GRASP55 delocalizes to the ER, autophagosome, multivesicular body or CUPS. Transmission electron microscopic images show that GRASP55 is localized at autophagosomes and multivesicular bodies under ER stress, mTOR inhibition, and starvation conditions (see text for details).

GRASPs are known to localize at the Golgi in the basal state, but changes in the phosphorylation (i.e., phosphorylation at S441 and dephosphorylation at T264 of GRASP55) and glycosylation (de-O-GlcNAcylation at the C-terminal region of GRASP55) status by certain stimuli can redirect their location to other sites such as the ER, autophagosomes, or MVBs, which leads GRASPs to perform other functions, particularly evident in UPS (Figure 2) (Kim et al., 2016; Noh et al., 2018; Zhang et al., 2018; Nüchel et al., 2021). It has been shown that phosphorylation at the C-terminal end region of GRASP55 by ER stress signals, particularly at S441, induces the ER redistribution of GRASP55 to facilitate the UPS of transmembrane proteins (Gee et al., 2011; Kim et al., 2016). In contrast, a recent study suggests that dephosphorylation at T264 by mTORC1 inhibition facilitates the movement of GRASP55 to autophagosomes and MVBs, enabling the UPS of selected cargos to reshape the extracellular proteome upon stress (Nüchel et al., 2021). Precisely how the phosphorylation at S441 and the dephosphorylation at T264 of GRASP55 evoked the same outcome of re-localizing GRASP55 into autophagy-associated structures needs to be further investigated.

An interesting observation in the electron microscopic analysis is that GRASP55 appears to be also localized on the inner vesicle membranes of autophagosomes and MVBs upon ER stress and mTORC1-inhibition (Noh et al., 2018; Nüchel et al., 2021) (Figure 2). According to the classical concept of the vesicle fusion process, cargos on the outer membrane of the MVBs, rather than on the membranes of the inner vesicles, would be suitably placed for localization to the plasma membrane, particularly for the type IV UPS of transmembrane proteins. In fact, a recent report has suggested that GRASP55 stays on the outer membrane of autophagosomes and surface of lysosomes under the starvation-induced conditions (Zhang et al., 2018). Further research is required to elucidate whether the outer membrane localization of GRASP55 is specific to starvation-induced UPS and how cargos on the membranes of the inner vesicles might reach the cell surface.

Although a number of studies have indicated the involvement of GRASPs in UPS, no consensus has yet been reached on a unified mechanistic role that they play in the process, with diverse features being reported depending on the experimental conditions (Dupont et al., 2011; Son et al., 2016; Zhang et al., 2018; Zhang et al., 2019; Liu et al., 2021). For example, it has been suggested that GRASP55 activates the early autophagy process but not its maturation, by showing that GRASP55 knockdown reduced the LC3-II levels and autophagosome formation in inflammation-associated IL-1β secretion (Dupont et al., 2011). In contrast, GRASP55 has been suggested to play a role in autophagosome maturation including fusion with lysosome via the physical interactions of GRASP55 with LC3 on the autophagosomes and with LAMP2 on the lysosomes under the starvation conditions (Zhang et al., 2018; Zhang et al., 2019; Liu et al., 2021). Outside these studies, there have been suggestions that GRASP55 principally mediates CFTR UPS as a cargo recruiting factor via a PDZ-based direct interaction with the cargo molecule under the ER stress-associated UPS conditions (Gee et al., 2011; Kim et al., 2016). In addition, GRASP55 knockout mice did not display discernible autophagy-related phenotypes in a study (Kim et al., 2020). Though precise mechanistic details are unknown at present, a common feature in these studies is that early autophagy components, but not those of late autophagy, contribute to the inflammation- or ER stress-associated UPS, while the late autophagy components as well as the early ones are involved in the starvation-related UPS.

### Ubiquitin

Another important molecule to be considered in autophagy-associated UPS is ubiquitin. Most proteins to be degraded via autophagy are ubiquitinated. Interestingly, several reports imply that deubiquitinated cargos are more prone to UPS as a means to evade autolysosomal or proteosomal degradation. For example, the ER-associated deubiquitinase USP19 (ubiquitin specific peptidase 19), which rescues the ERAD substrates ∆F508-CFTR and T-cell receptor-α (TCRα) from proteasomal degradation (Hassink et al., 2009), has been shown to play a critical role in the UPS of misfolded cytosolic proteins (Lee et al., 2016). In general, ESCRT-I components mediate the sorting of ubiquitinated cargo proteins to MVBs. MVB12B, an ESCRT-I component that does not have a ubiquitin-binding domain being different from other MVB12 proteins (Tsunematsu et al., 2010), has been shown to be indispensable in the rescue of ∆F508-CFTR via UPS (Noh et al., 2018). In light of the above, it is plausible that non-ubiquitinated substrates could be redirected to UPS with the assistance of ubiquitin-independent secretory structures while ubiquitinated ones go to disposal pathways via the canonical degradative process.

## Conclusion

It is evident that the UPS pathway intersects with autophagy-associated cellular mechanisms. For example, a number of ATG proteins and autophagy receptors/adaptors have been shown to be involved in the UPS of diverse cargo proteins. There is considerable diversity in each UPS process, however, depending on the cargo proteins involved. This begs several questions, requiring further investigation: 1) How are certain cytosolic and membrane proteins selectively incorporated into the UPS vesicles? 2) How are some proteins incorporated into autophagy-associated vesicles transported to the secretory pathway, with others following the degradation pathway? 3) Is deubiquitination required for cargos recruited to the UPS pathway evading the degradative route? 4) Are there mammalian structures corresponding to the yeast CUPS? 5) What could be regarded as the unified and prime role of GRASPs in UPS? and 6) Are intact lysosomes indeed required for the UPS of some cargo proteins?

Though the mechanisms of autophagy-related UPS are yet to be elucidated, there is mounting evidence of its applicability as a therapeutic target for human diseases. As abovementioned, autophagy-related UPS is largely involved in human diseases including neurodegenerative and metabolic ones (Kim et al., 2018; Gonzalez et al., 2020) and its clinical applications should be viewed as dependent on the specific disease-associated mechanisms involved. In the case of the UPS cargos whose excessive release provokes pathologies, such as IL-1β in diabetes and AD, α-synuclein in PD, and HMGB1 in sepsis, downregulation of their releases would be beneficial for disease control. On the contrary, activations of the UPS process could be employed as a therapeutic measure in some diseases, such as for FGF2 in AD and PD, IDE in AD, trafficking-deficient CFTR in cystic fibrosis, and trafficking-deficient pendrin in Pendred syndrome. For example, statins have yielded potential therapeutic results by inducing the autophagy-related UPS of IDE to relieve Aβ-induced pathologies in AD (Glebov and Walter, 2012; Son et al., 2015). Further investigation of the mechanistic details and pharmacological implications of UPS will provide the requisite knowledge to pave the way for the treatment of related diseases.

Each circumstance inducing UPS (e.g., starvation, ER stress, mechanical stress, developmental signals, etc.) should lead to varied cellular environments and structural organizations. Accordingly, it would be natural for each UPS substrate to be located under different conditions and its cell surface delivery to be engaged in different autophagy-related features. Furthermore, it is plausible that the same cargo protein could be delivered to the cell surface through dissimilar pathways dependent on the cellular circumstances as in the case of IL-1β and tau secretions (Merezhko et al., 2020). Despite substantial research being conducted, UPS still appears convoluted and requires future investigation for a more precise understanding of the diverse trafficking mechanisms which should, in turn, help the scientific community to discover potential ways to modulate the export of UPS cargos involved in human health and diseases.
